# Advancements in DNA methylation technologies and their application in cancer diagnosis

**DOI:** 10.1080/15592294.2025.2539995

**Published:** 2025-07-28

**Authors:** Yang Yang, Xiaosha Wen, Li Wang

**Affiliations:** a Laboratory Medicine Center, Shenzhen Nanshan District People’s Hospital, Shenzhen, PR China; b Department of Clinical Laboratory, The Affiliated Cancer Hospital of Zhengzhou University & Henan Cancer Hospital, Zhengzhou, China

**Keywords:** DNA methylation technology, cancer diagnosis, DNA methylation biomarkers

## Abstract

DNA methylation is a common epigenetic modification that maintains the integrity of the DNA sequence while profoundly influencing gene expression and phenotypic variation. Aberrant DNA methylation has been associated with the onset and progression of diseases, including cancer, metabolic disorders, and neurodevelopmental disorders. Recent advancements in detection technology led to a gradual increase in the exploration of DNA methylation as a valuable biomarker for cancer diagnosis and therapy. Single-base resolution has been achieved for whole-genome methylation analyses through second-generation sequencing technology, significantly enhancing detection efficiency. Additionally, PCR-based methods offer simple and feasible solutions for methylation analysis. In this review, we discuss various methods for detecting DNA methylation, focusing on bisulfite conversion-based techniques, methylation-sensitive restriction enzyme methods, enzyme conversion-based methods, third-generation sequencing approaches, and artificial intelligence. Furthermore, we briefly summarize the methylation biomarkers used for tumor diagnosis and the corresponding sample types employed. We believe that this information provides valuable insights for selecting and optimizing DNA methylation analysis tools.

## Introduction

DNA methylation plays a significant biological role and holds clinical application potential in cancer research [1–3], serving as a powerful diagnostic tool. Furthermore, investigating DNA methylation can help elucidate the mechanisms underlying tumorigenesis. DNA methylation primarily occurs at the 5 carbon position in cytosine residues, forming 5-methylcytosine (5mC) or 5-hydroxymethylcytosine (5hmC). In the human genome, DNA methylation occurs in regions containing CpG dinucleotides, with ~ 70–80% of these dinucleotides methylated [4]. The distribution of CpG sites is not uniform; instead, they tend to cluster, forming regions with elevated GC content known as CpG islands (CGIs), present in over 50% of gene promoters [5]. In humans, gene methylation primarily occurs in coding regions lacking CGIs, whereas promoter regions are typically unmethylated [6]. In contrast, during tumor formation, CGIs in promoter regions are highly methylated, leading to the transcriptional silencing or downregulation of gene expression. This process leads to the loss of tumor suppressor functions and subsequent genetic damage [7]. Consequently, there is increasing evidence that DNA methylation has substantial potential in early tumor diagnostics, treatment efficacy monitoring, and prognostic assessment [8–10].

Considerable progress has been made in methylation research due to the rapid development of detection technology [11,12]. Through innovation and improvement, scientists have successfully developed numerous efficient and accurate detection technologies that support methylation research. Among these methods, next-generation sequencing (NGS) platforms have proven to be highly effective in identifying methylation [13,14]. NGS provides more biological information than other approaches and enables the discovery of previously unknown methylation sites. Furthermore, the advancement of artificial intelligence has enabled researchers to apply machine- and deep-learning algorithms to develop single- or multimodal tumor diagnostic models using DNA methylation data [15–17]. By analyzing methylation sequencing data, these models identify specific methylation patterns associated with tumor initiation and progression, enhancing diagnostic sensitivity and specificity. This review focuses on the application of DNA methylation evaluation in tumor diagnostics and advanced technologies for assessing DNA methylation.

## Application of DNA methylation in cancer diagnostics

**Figure 1.**
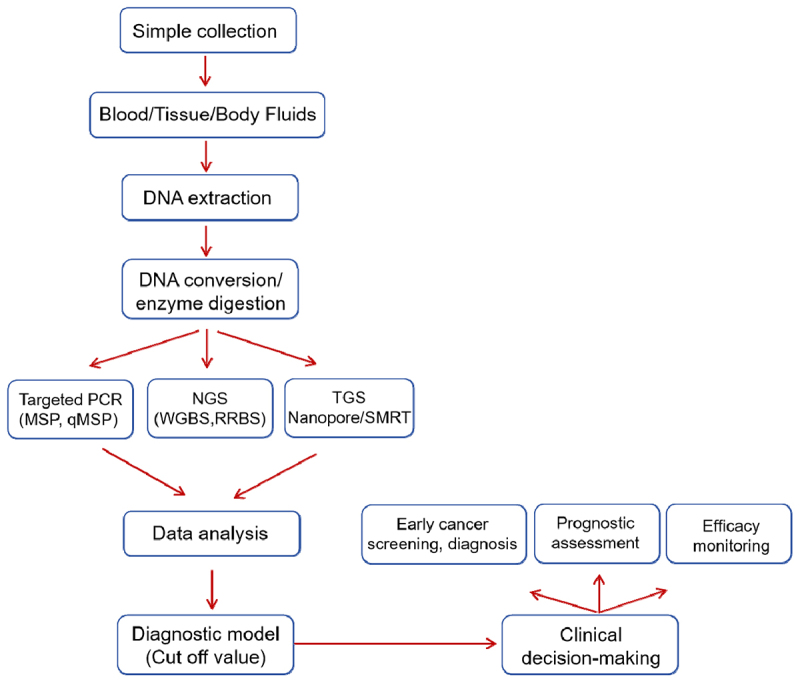
The workflow diagram for clinical decision-making using methylation biomarkers.

DNA methylation biomarkers are aberrant changes in DNA methylation in cancer cells and are considered promising indicators for cancer diagnosis during cancer development. It provides clinically actionable adjuncts to support tumor diagnosis and monitoring (Figure 1). Methods involving DNA methylation marker detection offer several advantages, such as early detection, precision, and traceability [18,19]. Many clinical studies have shown that, compared with traditional markers, DNA methylation markers exhibit superior sensitivity for early tumor screening and diagnosis [20,21]. Researchers can detect DNA methylation markers in different sample types, including tissue, plasma, urine, and sputum, to increase the sensitivity and specificity of tumor diagnosis. The choice of detection materials and biomarkers is important for the effective detection of DNA methylation in cancer diagnostics.

### Detection materials

The source of DNA methylation detection materials is crucial for ensuring the accuracy and reliability of the results. Tumor tissue is recognized as a reliable sample source, with biopsy results often regarded as the gold standard [22]. Researchers analyze DNA methylation in tumor tissue because it directly reflects the biological characteristics of tumor cells and provides essential information about their development and progression. Additionally, heterogeneity of tumor tissues enables the identification of methylation patterns at different tumor stages. Nevertheless, the tissue biopsy process is invasive and limited by factors such as tumor location and size [23]. Liquid biopsy is a noninvasive technology that can be used to diagnose cancer through the analysis of biomarkers in bodily fluids, such as blood, urine, and saliva [24,25]. This approach provides several advantages, including simple specimen acquisition, early-stage cancer diagnosis, and dynamic disease monitoring [26,27]. Peripheral blood is often used for liquid biopsies; analyzing circulating tumor DNA (ctDNA), circulating tumor cells, and extracellular vesicles enables the acquisition of critical cancer-related genetic information. Research findings have demonstrated that ctDNA is a highly desirable material for analyzing tumor DNA methylation [28–30]; however, the relatively low abundance of ctDNA in peripheral blood, especially in early-stage tumors, presents challenges for its extraction and detection [31]. Genomic DNA from peripheral blood mononuclear cells (PBMCs) can also be used for effective methylation detection and has been validated for the early diagnosis of breast and colorectal cancer, demonstrating improved sensitivity and specificity [32]. Samples from different sources should be explored to increase early diagnostic sensitivity.

Certain cancer types are a source of optimal sample materials. For example, methylation analysis of exfoliated cells from the cervix shows promise as an early cervical cancer diagnostic tool, potentially leading to increased sensitivity and accuracy [33]. Urine samples are noninvasive, resulting in high patient compliance with minimal interference [34]; these samples are particularly advantageous for detecting urinary system cancers, such as bladder and kidney cancer. Furthermore, fecal tumor gene testing is a promising screening method for colorectal cancer [35]. This approach involves the detection of methylation patterns and genetic mutations in exfoliated colorectal cells to evaluate colorectal cancer development risk. However, patient acceptance of fecal sample collection remains relatively low. Future efforts can focus on increasing the ease of the sample collection process and developing fully automated devices to separate fecal intestinal cells [36]. Overall, the selection of diagnostic materials aligned with distinct objectives is critical for the study of DNA methylation and the application of related tools. The use of bodily fluid samples represents a noninvasive detection method for early cancer screening and ongoing monitoring, enabling repeated sampling and improving patient acceptance. Liquid biopsy cannot replace tissue biopsy, as the sensitivity and specificity of liquid biopsy diagnostics are commonly lower than those of tissue biopsy. Furthermore, extensive genomic and gene expression profiling data can be obtained from tissue samples; these data are indispensable for accurately determining the type, grade, and molecular characteristics of tumors. In clinical practice, genomic and gene expression profiling methodologies can be integrated and tailored to the specific clinical context to obtain a more comprehensive and precise diagnostic assessment.

### DNA methylation biomarkers

As described above, the hypermethylation of tumor suppressor genes and the hypomethylation of oncogenes are critical in tumorigenesis. Methylation changes are observed in nearly all cancer types and occur during the precancerous or early cancer stages [37]. There has been an increasing focus on DNA methylation, accompanied by significant advancements in related scientific publications and clinical trials [3,38]. In particular, the exploration of novel methylation markers for the early diagnosis of tumors has attracted considerable attention. Utilizing high-throughput methylation detection technologies, researchers have analyzed various clinical samples to identify methylation markers with high sensitivity and specificity [39,40]. These markers have been further validated in clinical settings to assess their effectiveness and reliability in clinical practice. In one study, whole-genome bisulfite sequencing was employed to identify 15 optimal ctDNA methylation biomarkers for the early detection of breast cancer. In the validation cohort, these biomarkers demonstrated an area under the ROC curve of 0.971 [41]. A prospective cohort study, ColonSecure, focused on detecting DNA methylation levels via cfDNA, evaluating approximately 3,500 individuals at high risk for colorectal cancer (CRC). ColonSecure successfully identified 89 out of 103 patients diagnosed with CRC through colonoscopy, demonstrating a sensitivity of 86.4% and a specificity of 90.7% [42]. This validation study also revealed that compared with conventional serum markers (CEA, CRP, and CA19–9), the identified methylation sites were more sensitive for diagnosing early CRC. Another study assessed featured DMCs between esophageal squamous cell carcinoma (ESCC) and matched adjacent normal tissues, identifying 35,577 DMCs with a 450K microarray. Furthermore, researchers have established a panel of 12 methylated CpG sites that can clearly distinguish ESCC from normal tissues. When this panel was evaluated using TCGA ESCC data, the area under the curve was 96.6% [43]. Wang T et al. investigated DNA methylation levels in PBMCs derived from individuals with breast cancer and controls [44]. Four unique methylation biomarkers were identified, demonstrating a diagnostic sensitivity of 93.2% and a specificity of 90.4%, which are superior to those of conventional markers. We have summarized the DNA methylation markers for the early diagnosis of different cancer types, including specific cancers and their corresponding methylation sites, in Table 1 [45–64].

**Table 1.** DNA methylation biomarkers in cancer diagnosis.

Cancer	Methylation biomarkers	Sample type	Detection method	References
Lung Cancer	SHOX2, RASSF1A,PTGER4	Tissue、Blood、Bronchoalveolar lavage fluid	Methylight，NGS	[45,46]
Hepatocellular carcinoma	SEPT9, BMPR1A, PLAC8	Tissue、Blood	BSP	[47,48]
Gastric cancer	RNF180、SEPTIN9	Tissue、Blood (plasma)	Methylight	[49]
Colorectal cancer	SDC2、SFRP2、SEPT9	Tissue、Feces、Blood	Real-time PCR with fluorescent probe	[50–52]
Breast cancer	TRDJ3、PLXNA4、KLRD1、KLRK1	PBMC、Tissue、Blood	Pyrosequencing、Targeted bisulfite sequencing、Real-time PCR with fluorescent probe	[29,44]
Cervical cancer	DPP6、RALYL、GSX1、PCDHGB7	Cervical smear	Real-time PCR with fluorescent probe	[53,54]
Renal cell carcinoma	OXR1, MST1R	Tissue、Blood and Urine	qMSP、NGS	[55,56]
Bladder cancer	CFTR、SALL3、 TWIST1	Urine	Pyrosequencing	[57]
Pancreatic cancer	PRKCB、KLRG2 ADAMTS1、BNC1	Tissue、Blood	WGBS、Real-time PCR with fluorescent probe	[58,59]
Esophageal cancer	OTOP2、KCNA3	Tissue、Blood	Real-time PCR with fluorescent probe、WGBS	[60,61]
Ovarian cancer	COL23A1、C2CD4D、WNT6	Tissue、Blood	RRBS、Targeted bisulfite sequencing	[62]
Prostate cancer	CHST11、CUGBP2、PCDHGC4	Tissue、Blood	WGBS、MSRE	[63]
Glioblastomas	MGMT	Tissue	Real-time PCR with fluorescent probe	[64]

## Detection methods

**Figure 2.**
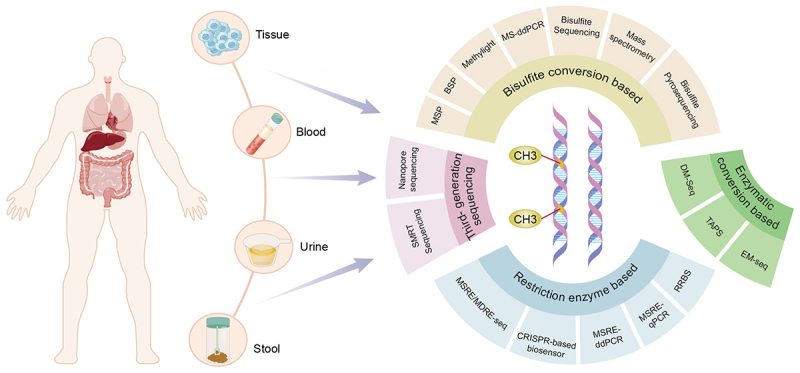
The materials and techniques used for detecting DNA methylation. There are four aspects: bisulfite modification, restriction enzyme-based, enzymatic conversion, and third-generation sequencing.

Accurate methylation detection techniques play a vital role in tumor diagnosis. Here, we systematically summarize four primary groups of techniques for identifying methylation sites: 1) bisulfite-converted DNA-based methods, 2) MSRE-based approaches, 3) enzyme conversion-based methods, and 4) third-generation sequencing methods (Figure 2).

### Bisulfite-converted DNA-based methods

**Figure 3.**
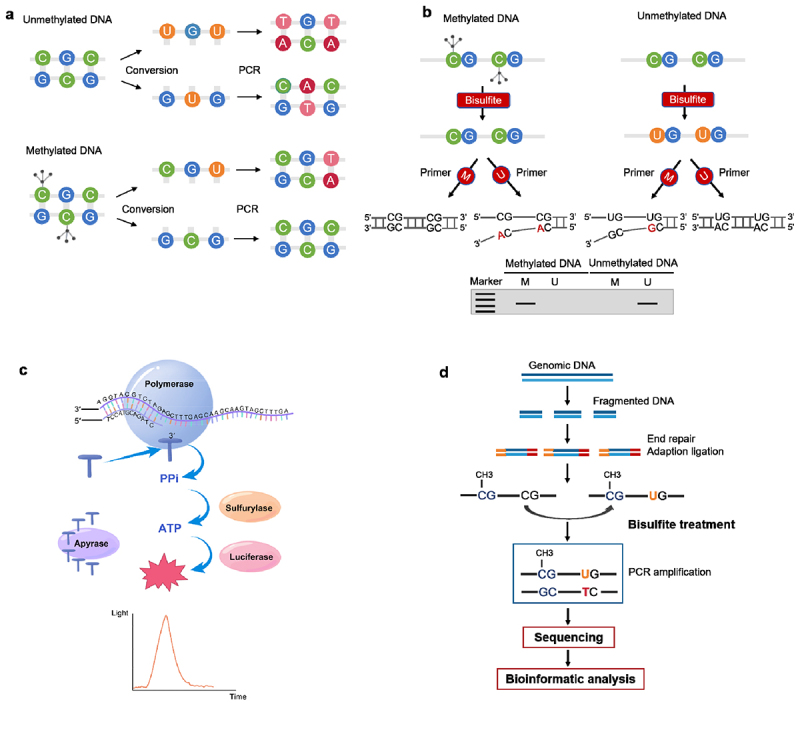
Common techniques based on bisulfite conversion. a: schematic diagram of the principle of bisulfite conversion; b: schematic diagram of the BSP methylation detection technique; c: schematic diagram of pyrosequencing methylation detection; d: the working principle of bisulfite sequencing for detecting DNA methylation.

Bisulfite conversion is considered the gold standard for determining DNA methylation [65]. Currently, bisulfite conversion-based methods, such as methylation-specific PCR, bisulfite sequencing, and pyrosequencing, are the most widely used. Upon bisulfite treatment, unmethylated cytosines (C) in CpG dinucleotides are converted to uracil (U), whereas methylated C remains unchanged (Figure 3a), enabling molecular differentiation between these two states. Bisulfite conversion kits, such as the EpiTect Bisulfite Kit (QIAGEN, 98.7%) and EZ DNA Methylation-Direct Kit (Zymo Research, 99.9%), exhibit high conversion rates and have demonstrated effective results [66].

#### PCR-based methods

Methylation-specific PCR (MSP) is a commonly used technique for detecting DNA methylation status. Two sets of primers are designed: one specific for methylated sequences and the other for unmethylated sequences [67]. These primers selectively amplify either methylated or unmethylated loci within the target sequence. The PCR product is analyzed via electrophoresis to determine whether the amplified fragments are methylated at specific sites. MSP can also detect multiple methylation sites for targeted fragment analysis (Figure 3b). The Methylight method derived from MSP can be utilized for methylation analysis using primers and probes that specifically target methylated fragments [68]. The level of methylation is assessed by comparing the differences in CT values relative to an internal control. This method does not require gel analysis but does necessitate the use of standards as controls. Some methylation detection kits have been approved by the FDA, such as the HCCBloodTest for hepatocellular carcinoma and the EpiproLung test for lung cancer, indicating that Methylight has significant application value [69].

Methylation-specific droplet digital PCR (MS – ddPCR) can be employed for methylation analysis [70,71]. ddPCR provides high sensitivity and allows for absolute quantification; thus, it is promising for molecular detection applications. MS – ddPCR involves designing primers and probes specific to methylated fragments. The DNA fragments undergo bisulfite conversion and subsequent emulsification, creating small droplets that each contain a single DNA molecule. Upon amplification, these droplets emit fluorescence, facilitating the identification of methylation sites. MS – ddPCR is highly sensitive and enables absolute quantification [72]. Furthermore, some isothermal amplification techniques, such as loop-mediated isothermal amplification (LAMP) or rolling circle amplification, are also efficient and cost-effective means for methylation detection [73–75]. Methylation detection methods derived from amplification techniques combined with bisulfite conversion are generally simple to apply and offer short processing times and better sensitivity and specificity. Nevertheless, these methods are limited in the methylation sites they can detect and are constrained to known sites. As a result, these methods are only feasible for detecting specific cancer types.

#### Sequencing-based methods

Sequencing-based methods typically detect single-base differences and can reveal new differential methylation sites. Sanger sequencing offers the advantages of long read lengths (1,000 bp) and high accuracy; thus, this technique is widely applicable. The amplification products of bisulfite-treated DNA can be analyzed with Sanger sequencing; however, this method has low sensitivity. Bisulfite sequencing PCR (BSP), the classic method for detecting DNA methylation [76,77], involves amplifying target DNA fragments treated with bisulfite, followed by purifying the PCR products and cloning them into TA vectors to perform sequencing. Finally, the obtained sequences are aligned with the unmodified original sequences to quantify the number of methylation sites and their methylation level. BSP enables quantitative studies and exhibits high sensitivity; however, it requires extensive cloning screening, which is relatively complex and time-consuming. The accuracy of quantification is limited by the number of clones.

Bisulfite pyrosequencing has high accuracy, similar to that of Sanger sequencing [78], but with a more rapid sequencing speed and higher throughput (Figure 3c). Pyrosequencing is regarded as the gold standard for methylation detection, enabling both qualitative and quantitative analyses of methylated sites [79]. This method relies on the detection of released pyrophosphate during DNA synthesis to determine the DNA sequence. When bisulfite-treated DNA is recognized by primers, the DNA polymerase incorporates the correct nucleotides into the synthesized DNA strand. This process releases pyrophosphate from dNTPs, triggering a series of enzyme-catalyzed reactions. Finally, these reactions generate light signals, allowing the instrument to collect the DNA sequence information. Pyrosequencing is simple to perform and exhibits better reproducibility for detecting genetic variations in specific genes [80]. However, its throughput is comparatively lower than that of NGS platforms, rendering it unsuitable for large-scale whole-genome sequencing endeavors.

The NGS platform is a mainstream sequencing technology for detecting DNA methylation. Bisulfite sequencing is widely used to detect 5mC and 5hmC [81–83]. It is relatively mature, with many related test kits available, enabling the precise evaluation of DNA methylation levels. The development and application of DNA methylation microarrays are one of the essential components of high-throughput methylation sequencing. The microarray is equipped with a vast number of oligonucleotide probes. For instance, the commonly used 450k microarray contains 450,000 probes, which are capable of binding to 450,000 CpG sites [84]. The newly developed 850k microarray has the capacity to detect an even greater number of CpG sites, up to 850,000. The probes are categorized into two types: Type I and Type II. Type I probes consist of pairs, each containing a methylation-specific probe and an unmethylation-specific probe. In contrast, Type II probes are single probes, with their 3’ ends positioned on one or more CpG sites of interest. Through different single-base extension reactions, the presence of C or T at these sites can be simultaneously detected. The dominant platform for DNA methylation microarrays is Illumina, particularly the Illumina Infinium EPIC 850K, which is currently the gold standard for investigating DNA methylation changes at specific CpG sites across the whole genome in large scale studies [85].

Whole-genome bisulfite sequencing (WGBS) has been used to analyze DNA methylation across the entire genome. This approach enables the generation of a genome-wide methylation profile, revealing methylation changes associated with various disease states. Therefore, the application of WGBS offers significant potential for the early detection of cancers [86,87]. The current technical workflow of WGBS typically comprises five steps: sample preparation, library construction, bisulfite treatment, sequencing, and bioinformatics analysis (Figure 3d). Some kits necessitate library construction prior to bisulfite conversion. WGBS is especially well suited for comprehensive pancancer screening applications, providing precise methylation information at single-base resolution. However, the cost of WGBS is relatively high, as it involves sequencing and analyzing the entire genome. Additionally, WGBS generates large quantities of data, increasing the complexity of the analysis. Targeted sequencing has emerged as a feasible alternative to WGBS for targeted methylation detection [88–90]. The SeqCap Epi CpGiant Enrichment System, developed by Roche NimbleGen, is a high-performance targeted bisulfite sequencing technology for in-depth DNA methylation analysis within specific genomic regions [91], such as CpG islands and promoters. The workflow of this system encompasses three key steps: bisulfite conversion, customized target probe hybridization capture, and NGS. It can achieve a sequencing depth of > 100X, offers customization options, is well suitable for studies with limited sample sizes. By reducing the scope of sequencing, targeted sequencing can also reduce costs while still providing valuable methylome data. Notably, the NGS platform for DNA methylation analysis is subject to the limitation of read length (150–300 base pairs), which may affect coverage and resolution in complex genomic regions. Bisulfite sequencing also demands high-quality input DNA samples, and the fragmentation of cfDNA imposes even stricter requirements on the sequencing process [92]. Overall, It is a fundamental method for studying DNA methylation patterns that relies on the conversion of unmethylated C to U via bisulfite treatment. Despite its widespread application, Bisulfite sequencing presents several limitations [93,94]. First, the bisulfite conversion process causes DNA depurination, resulting in fragmentation that complicates the long-read sequencing performance. Second, converting unmethylated C to T decreases the sequence complexity of the bisulfite-treated library, thus resulting in uneven genome coverage.

#### Mass spectrometry analysis

Mass spectrometry is an analytical technique used to detect DNA methylation that can distinguish and quantify DNA methylation with high sensitivity and precision. Commonly used mass spectrometry techniques include matrix-assisted laser desorption/ionization time‒of-flight mass spectrometry (MALDI – TOF) and liquid chromatography‒mass spectrometry (LC‒MS) [95,96]. The MassARRAY system combines MALDI – TOF with bisulfite treatment, wherein DNA is treated with bisulfite, followed by PCR amplification of specific DNA regions [97]. The mass differences of the amplified products are detected via mass spectrometry, allowing inference of the degree of DNA methylation. MassARRAY can detect extremely low concentrations of nucleic acid fragments [98] and multiple targets simultaneously in a short assay time. The requirement for specific mass spectrometry instrumentation limits its application in laboratories.

In response to the challenges associated with bisulfite treatment, researchers are working to improve and optimize the process of DNA methylation conversion as follows. 1) Reducing the conversion reaction time: An innovative approach has been developed that integrates ammonium bisulfite with sodium bisulfite, increasing the DNA denaturation temperature to 98°C for 10 minutes. Ultrafast BS sequencing effectively minimizes DNA damage and background noise, resulting in a lower false-positive rate than that achieved with conventional BS sequencing. 2) Increasing DNA recovery after conversion: Employing novel purification techniques with column chromatography or magnetic beads can reduce DNA loss during the purification steps. For example, the EZ DNA Methylation-Gold™ Kit, produced by Zymo Research, utilizes an innovative column-based desulfation technology that enables the simultaneous purification and desulfation of DNA, minimizing unnecessary DNA precipitation.

### MSRE/MDRE-based assays

Methylation-sensitive/-dependent restriction enzymes (MSREs/MDREs) are a class of restriction enzymes whose activities are dependent on the methylation status of DNA. MSREs selectively cleave the unmethylated DNA sequence at specific sites. However, no activity is observed on methylated DNA, such as HpaII (CCGG), BstUI (CGCG), and SmaI (CCCGGG). Pairs of MSREs, such as HpaII and MspI, exhibiting distinct sensitivities to a given restriction site are commonly employed for analysis [99]. HpaII is sensitive to DNA methylation and cannot cleave methylated CG sites, whereas MspI cuts the DNA at the restriction site without being affected by the methylation status. Conversely, MDREs, such as MspJI (mCNNRN9) and LpnPI (CmCDGN10), which are commonly integrated with NGS, specifically cleave methylated DNA sequences [100]. MSRE/MDRE-based assays enable the detection of methylation without conversion, which offers advantages such as simplicity and minimal DNA damage [101]. These assays provide an alternative to bisulfite conversion methods and minimize DNA damage.

#### PCR-based methods

**Figure 4.**
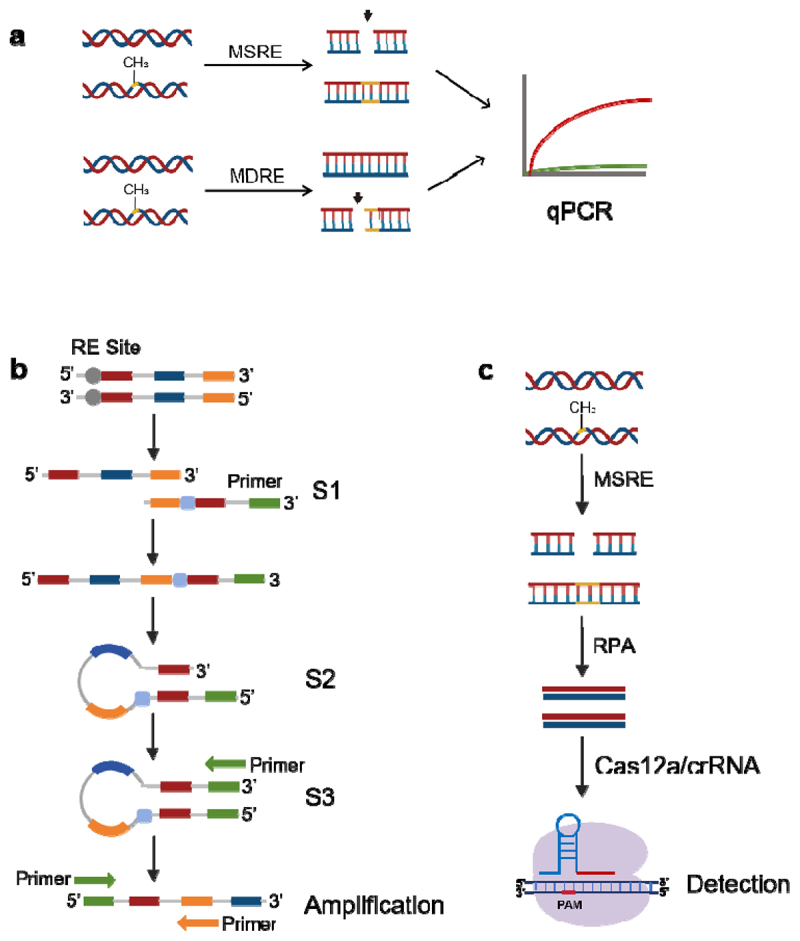
Methylation detection methods related to MSRE/MDRE. a: schematic overview of MSRE/MDRE qPCR; b: the mechanism of STEM-PCR locus-specific detection; c: schematic of MeCRISPR detection of DNA methylation. MSRE, methylation-sensitive restriction enzyme. MDRE, methylation-dependent restriction enzymes. RE, restriction enzyme. S1, segment 1. S2, segment 2. S3, segment 3. RPA, recombinase polymerase amplification.

The MSRE-based PCR method is a conventional approach for analyzing DNA methylation [102,103]. MSREs are used to digest unmethylated fragments while preserving methylated fragments for subsequent PCR or qPCR amplification to determine the methylation status of targeted DNA regions (Figure 4a). These methods are efficient and rapid; however, they exhibit relatively lower sensitivity than other methods. Recent studies have outlined the development of more sensitive methylation detection technologies that combine MSRE/MDRE with PCR. Xu G et al. developed a specific terminal-mediated PCR technique based on MDREs [104]. The method exhibited a sensitivity of up to 0.1% and a detection limit of 5 copies per reaction. This method involves the recognition of specific methylated sites via MDRE, leading to the excision of the S1 sequence and the generation of a novel 5’ end. The initial round of PCR amplification utilized the S3 primer, which contained a sequence complementary to the S2 sequence. This led to intramolecular base pairing, causing the formed S3 sequence to adopt a loop structure with a 3’ overhang and enabling further extension during subsequent amplification. In contrast, unmethylated fragments lacking cleavage by MDRE cannot be amplified because of the S1 sequence at the 3’ end of the S3 primer. Thus, this approach ensures the specific amplification of methylated segments (Figure 4b). In addition, researchers have combined other amplification techniques with MRSE to analyze methylation. For example, MSRE – ddPCR was developed to detect DNA methylation levels. This method offers greater sensitivity, allowing for the detection of low amounts of DNA (as low as 0.651 ng) within a range of 0.1% to 0.01% [105]. Additionally, Tan YL et al. utilized LAMP combined with MSRE to detect methylation in the DAPK1 gene associated with cervical cancer [106], achieving a sensitivity of 1%, with a limit of detection (LOD) of 5 aM (equivalent to 3 copies/μL), or 0.01%, with an LOD of 250 aM. This method provides isothermal amplification without the need for complex equipment. Overall, PCR-based assays provide an effective tool for the rapid detection of specific methylated targets and sites.

#### CRISPR-based biosensors

Recent advancements in methylation detection have been made using the CRISPR/Cas system [107–109]. Cas proteins, acting as nucleases, precisely cleave target sequences in the direction of sgRNA/crRNA, ensuring high specificity. Among the Cas proteins, Cas12, Cas13, and Cas14 can nonspecifically cleave DNA or RNA fluorescent reporters upon activation of their enzymatic cleavage function, resulting in the emission of fluorescent signals [110,111]. Notably, the ability of the CRISPR/Cas system to amplify signals enhances the sensitivity of nucleic acid detection. Xu W et al. developed a rapid and highly sensitive methylation detection method using MSREs combined with the RPA-based CRISPR/Cas12a system (MeCRISPR) [112] (Figure 4c). MSREs selectively cleave nonmethylated DNA fragments but exhibit no enzymatic activity toward methylated DNA. Methylation-modified DNA sequences can subsequently be amplified via RPA and then detected via the CRISPR/Cas12a system, which produces fluorescent signals. This method has an LOD of 1 copy/µL with a sensitivity of 0.01%. When this method was applied to detect SEPT9 gene methylation in exfoliated cervical cells from 53 patients with cervical cancer and controls, the sensitivity and specificity were 100% and 92.3%, respectively. Han ZW et al. utilized the CRISPR/Cas12a system combined with transcriptional amplification to detect DNA methylation [113]. After bisulfite conversion, the methylated C remained unchanged, completely matching probes T and P, whereas the unmethylated C was transformed into U, causing mismatches. Owing to the specificity of T4 DNA ligase, only single-stranded nicks fully base-paired to complementary DNA double strands can be ligated. The ligated fragment then facilitates the synthesis of crRNA via the T7 promoter, which subsequently activates the nuclease function of Cas12a, leading to the emission of a fluorescent signal within the detection system. This method achieves a detection limit of 337 aM and can distinguish methylation levels as low as 0.01%; thus, it is effective for detecting methylation in the genomic DNA of cancer cells.

CRISPR-based biosensor methods are known for their high sequence specificity and signal amplification, demonstrating better sensitivity and specificity than other methods in detecting DNA methylation [114–116]. These methods offer quick detection times and reduce reliance on complex equipment. Nevertheless, the high sensitivity of biosensors can increase their susceptibility to signal disruptions, which may result in false positives. The integration of DNA amplification within CRISPR biosensors results in a lower detection limit. In contrast, nonamplification-based methods generally have higher detection limits and need more sample input. Additionally, the design of sgRNA/crRNA mandates specific sequence requirements, such as the presence of a PAM site. Therefore, it is important to develop additional Cas proteins that alter PAM specificity to expand the range of targets.

#### MSRE/MDRE-based sequencing

MSRE/MDRE-based NGS (MSRE/MDRE-seq) methods are also effective in profiling whole-genome DNA methylation landscapes [117,118]. The analytical workflow generally integrates restriction enzyme digestion, adaptor ligation, size selection, PCR amplification, and sequencing. After sequencing, a computational pipeline is utilized to refine fragment selection and reduce noise through a CG filter, followed by sequence alignment and identification of differentially methylated regions. Reduced representation bisulfite sequencing (RRBS) is a cost-effective method that requires a low sample input (10–300 ng) to analyze DNA methylation patterns, making it feasible for cfDNA samples [119,120]. This approach uses the MspI enzyme to cleave DNA at CCGG sites independent of methylation status. The resulting fragments are ligated with adaptors and bisulfite treated, and subsequently enriched CpG sites are sent for sequencing. MDRE-seq can also be used to effectively analyze the DNA methylation status. One study demonstrated that LpnPI digestion combined with NGS provides highly reproducible whole-genome CpG methylation profiles for more than 50% of potential methylated CpGs with a sequencing depth of less than one-tenth of that required for WGBS [121]. MspJI-seq can identify hemimethylation sites with accuracy comparable to that of bisulfite sequencing [122]. MSRE/MDRE-seq offers a high-throughput alternative for 5mC and 5hmC analysis.

Methods for detecting methylation using MSRE/MDRE demonstrate high sensitivity in identifying specific methylation sites. These procedures are relatively simple and expedited, avoiding the DNA damage caused by the methylation conversion process. Nevertheless, these endonucleases possess sequence specificity and cannot recognize the full spectrum of methylation sequences, imposing certain limitations on their application. Consequently, MSRE/MDRE-based methylation techniques are applicable for identifying distinct methylation regions associated with specific cancers, enabling the development of targeted assay kits for efficient tumor screening programs.

### Methods based on enzyme-converted DNA

Enzymatic transformation methods are highly valuable for epigenetic sequencing, as they are highly accurate and cause minimal DNA damage [123]. Enzymatic conversion technologies are primarily integrated with NGS, as this review introduces several common enzymatic approaches for methylation detection. These methods commonly utilize a two-step enzymatic conversion process.

#### EM-seq

**Figure 5.**
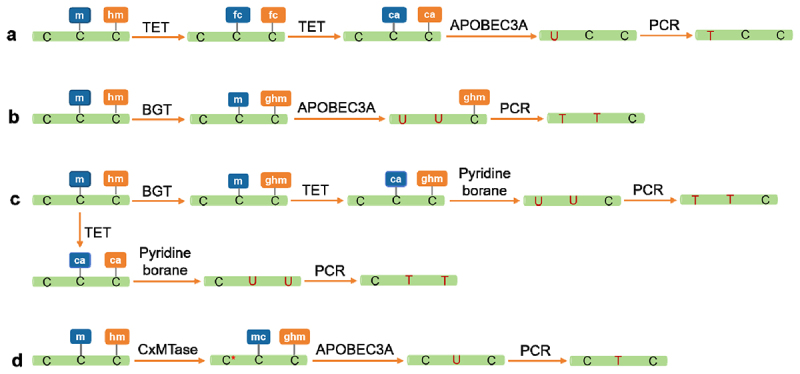
Enzymatic transformation methods for detecting DNA methylation. a: the working principle of EM-seq technique for detecting 5mc and 5hmc. b: schematic diagram of EM-seq technique for detecting 5hmc. c: schematic of TAPS detection of 5mc and 5hmc; d: the principle of DM-Seq technique for detecting 5mc. EM-seq, enzymatic-based DNA methylation sequencing. DM-Seq, direct methylation sequencing. TAPS, TET-assisted Pyridine Borane sequencing.

Enzymatic-based DNA methylation sequencing (EM-seq), as an alternative to bisulfite sequencing, minimizes DNA damage to enable the generation of methylome libraries with superior quality that provide accurate methylation information from fewer sequencing reads [124,125]. This approach involves two types of enzymes: methylcytosine dioxygenase (TET) and apolipoprotein B mRNA editing catalytic polypeptide-like 3A (APOBEC3A). Initially, TET is used to oxidize 5mC and 5hmC into 5caC. Subsequently, APOBEC3A deaminates unmethylated C to U, whereas 5caC remains unaffected. Consequently, EM-seq yields the same sequence as bisulfite treatment but better preserves DNA integrity during sequencing, enhancing the coverage of sequencing data (Figure 5a). Moreover, EM-seq requires minimal DNA input, with thresholds as low as 10 ng, making it compatible with cfDNA samples [126]. The TWIST Human Methylation Panel, co-developed by Twist Bioscience and New England Biolabs®, is based on the EM-seq™ technology platform and has become one of the predominant high-throughput methylation sequencing solutions widely adopted in genomic applications [127]. Many studies have also demonstrated that EM-seq provides high resolution for cfDNA methylation analyses [17,128]. The differential methylation sites identified contribute to the establishment of early tumor diagnosis models with high sensitivity and specificity. In addition, integrating APOBEC3A with T4 β-glucosyltransferase (T4-BGT) enables the differentiation of 5mC and 5hmC (Figure 5b) and the exploration of new biomarkers for cancer diagnosis and therapy. Since EM-seq still converts unmethylated C into T, it causes a base imbalance in the methylation sequencing library, reducing the efficiency of sequencing data utilization.

#### TAPS

The TET-assisted Pyridine Borane Sequencing (TAPS) technique, in contrast to EM-seq, uses a forward conversion approach, directly converting 5mC into T [129,130]. This approach uses the TET enzyme to oxidize 5mC and 5hmC into 5caC. 5caC is subsequently reduced by pyridine borane to dihydrouracil (DHU), which can be converted into T during PCR amplification (Figure 5c). TAPS can also distinguish between 5mC and 5hmC [131]. TAPS utilizes mild reaction conditions at room temperature, which minimizes damage to DNA fragments and preserves longer sequences, up to 10 kb. As a result, libraries constructed with TAPS show increased complexity and enhanced sequencing quality. Moreover, TAPS effectively converts methylated C to T, reducing base composition bias. When integrated with third-generation sequencing platforms, TAPS further improves the accuracy of sequencing outcomes [132]. Research has shown that TAPS requires less cfDNA for methylation library construction and yields high-quality data on methylation sites [133]. Although whole-genome TAPS (wgTAPS) effectively reduces sequencing costs, the cost of TAPS remains relatively high. Researchers developed reduced representation TAPS (rrTAPS), a simplified technique for detecting CpG-rich regions of the genome. This process involves the digestion of the genome with the MspI enzyme before TAPS conversion and subsequent library preparation and sequencing [134]. Owing to the restriction sites of the MspI enzyme, the number of CpG sites captured by rrTAPS is limited. Researchers have developed the endonuclease enrichment TAPS (eeTAPS) technology, where genomic DNA is first subjected to TAPS to convert mCpG to DHU sites and then digested with uracil-specific excision enzyme before library preparation and sequencing. This approach enables the enrichment of mCpGs across the entire genome, offering a cost-effective solution and a novel approach for genome-wide sequencing.

#### DM-Seq

A new bisulfite-free method, direct methylation sequencing (DM-Seq), has recently been developed for mapping 5mC at the single-base level [135,136]. This method can also discern the difference between 5mC and 5hmC. DM-Seq involves two DNA-modifying enzymes: DNA methyltransferase and DNA deaminase. Researchers engineered a methyltransferase fused with an S-adenosyl methionine analog to inhibit the deamination activity of the A3A enzyme (Figure 5d). Employing the fusion enzyme on the DNA template, the unmethylated C is modified to *C, and 5hmC is modified to 5ghmC; however, 5mC remains unchanged. Subsequent treatment with the A3A enzyme results in *C remaining as C, 5ghmC reverting to C, and only 5mC deaminating to T. Therefore, DM-Seq directly sequences 5mC, allowing for the precise sequencing-based localization of methylation. Furthermore, DM-seq requires significantly less DNA input than BS-seq does. As an emerging method for DNA methylation analysis, DM-Seq technology requires further optimization of the conversion conditions to increase the conversion efficiency. Enzymatic conversion methods generally involve a two-step process, making their stability and conversion efficiency susceptible to various factors. Future research should be conducted to optimize the reaction conditions, streamline the procedures, and improve the conversion efficiency for broader applications in methylation detection.

### Third-generation sequencing

Third-generation sequencing (TGS), known as single-molecule sequencing, primarily includes single-molecule real-time (SMRT) sequencing, nanopore sequencing, and Helicos single-molecule sequencing [137,138]. Among these, SMRT and nanopore sequencing have been developed for their potential in DNA methylation analysis. TGS enables the direct identification of DNA methylation without additional chemical modifications or labeling steps, with the advantages of high throughput, long read lengths, and the ability to perform single-molecule and real-time sequencing. Unlike the short read lengths of NGS (150–300 bp), TGS can achieve read lengths on the scale of 1 Mb [139]. In addition, TGS does not require PCR amplification, thus avoiding potential biases and errors introduced during the amplification process. These merits make TGS platforms highly promising for applications in epigenetics, particularly in methylation sequencing.

#### Nanopore sequencing

**Figure 6.**
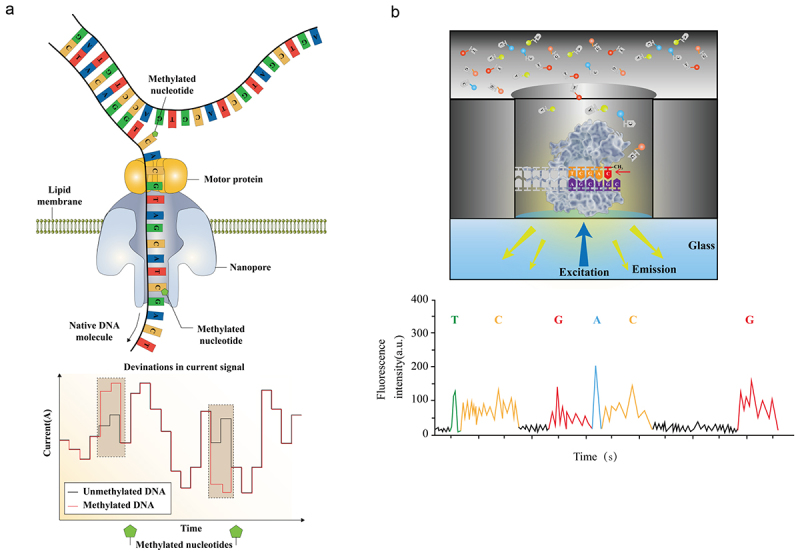
The principle of of nanopore sequencing and SMRT sequencing in detecting DNA methylation. a: schematic diagram of nanopore sequencing for detecting DNA methylation; b: schematic overview of SMRT sequencing in detecting DNA methylation. SMRT, single molecule real-time.

Nanopore sequencing, developed by Oxford Nanopore Technologies, identifies bases by measuring the electrical current changes caused by individual nucleotides as they pass through a nanopore [140] (Figure 6a). The fine diameter of the nanopore allows only single nucleic acid polymers to pass through. When a DNA or RNA molecule traverses a nanopore, bases with distinct electrochemical properties can alter the local current. These changes are recorded by sensors and converted into sequence information. If the C is modified with methylation, it will generate a distinct electrical signal compared with the unmodified C, thus revealing the DNA modification status. Nanopore sequencing technology has been applied to detect tumor DNA methylation. In one study, researchers utilized nanopore sequencing to sequence cfDNA in the cerebrospinal fluid of patients with brain tumors. By employing a random forest classifier to analyze copy number variations and overall DNA methylation patterns, the detection rates for brain tumors reached 88% and 45%, respectively [141]. Lau BT et al. developed a model employing nanopore-based single-molecule sequencing to detect methylation in cfDNA, which increased the sensitivity of ONT – cfDNA detection by an order of magnitude [142]. This model, when applied to the methylation profiling of cfDNA from colorectal cancer patients, revealed stark differences in methylation levels between affected individuals and healthy controls. The portable ONT platform, which does not require complex sample processing steps or PCR amplification, offers a distinct advantage in its rapid turnaround time [143], enabling real-time detection and holding substantial potential for clinical application. Despite its many advantages, challenges remain with nanopore sequencing in terms of the accuracy of DNA methylation sequencing, primarily due to the subtle difference in electrical signals produced by 5mC and unmodified cytosine, making them difficult to distinguish. The accuracy of 5mC detection can be improved by optimizing signal processing algorithms and increasing the resolution of nanopore devices. Researchers have developed a novel DeepMod algorithm for analyzing nanopore sequencing data with a detection accuracy of 99% for 5mC, reaching single-base resolution across the entire genome [144]. Furthermore, the development of the deep-learning algorithm Rockfish has significantly improved the detection capability of 5mC using ONT [145]. Additionally, advancements in the architecture of nanopores and the engineering of motor proteins have demonstrated the potential to increase the precision of base modification recognition [146,147]. Given that the base-calling inaccuracies in nanopore sequencing are stochastic and exhibit no base specificity, implementing iterative sequencing strategies facilitates the rectification of these discrepancies.

#### SMRT sequencing

SMRT sequencing, developed by the Pacific Biosciences company, differs from nanopore sequencing in its principle of operation (Figure 6b). It utilizes a ‘sequencing by synthesis’ approach, allowing real-time observation of the DNA polymerase as it synthesizes the DNA strand [148]. When the SMRT chip is used as a platform, the amplification reaction occurs within zero-mode waveguides (ZMWs). The diameter of the ZMW is smaller than the laser wavelength, which restricts the propagation of the excitation light. Only the fluorescence generated by chemical reactions within the ZMW can be detected, reducing background noise and enhancing signal accuracy. When DNA polymerase incorporates fluorescently labeled nucleotides into the growing DNA strand, the signals are captured and recorded. The methylation of C can decrease the rate of polymerase elongation, leading to increased separation between adjacent peaks. Therefore, SMRT sequencing is utilized for the detection of methylation patterns. This real-time monitoring allows SMRT technology to directly observe the incorporation of individual nucleotides and decode the DNA sequence. SMRT sequencing is known for its rapid sequencing speed, averaging 10 bp/s; however, it has a relatively high base-calling error rate [149]. The holistic kinetic model developed by the Lu team analyzes C methylation sites by assessing the dynamic signals of the DNA polymerase in conjunction with the sequence context with SMRT sequencing. By utilizing the methylation classification model trained by the convolutional neural network, SMRT sequencing demonstrated 90% specificity and 94% sensitivity in detecting 5mC methylation [150]. Moreover, SMRT sequencing can improve sequence consistency by increasing sequencing coverage and repetitive detection [151]. SMRT sequencing has been applied in various research fields [152–154]; however, there is still a need for optimization and improvement of the technological platform.

In recent years, the rapid development of TGS technology has significantly transformed the landscape of genomic research and molecular diagnostics. In the field of tumor molecular diagnostics, TGS has demonstrated the ability to accurately identify key biomarkers within the genome, such as structural variations, methylation patterns, and copy number variations, indicating its potential for widespread application. We have reviewed the sample requirements of various sequencing companies and concluded that solid tissues are the optimal choice for TGS methylation studies. Among these, fresh tissues and frozen (Fresh > frozen > FFPE) are the preferred option. There are several areas that require improvement: 1) enhancing detection accuracy, particularly for methylation; 2) increasing the detection sensitivity while reducing the amount of sample input; 3) increasing sequencing throughput; and 4) reducing costs and improving bioinformatics tools for data analysis.

### Artificial intelligence

#### Machine learning

Artificial intelligence (AI) is an interdisciplinary field of computer science focused on enabling machines to perform tasks requiring human intelligence. AI technology has demonstrated significant potential and value in various fields, such as disease diagnosis, medical image recognition, and drug discovery [155]. Machine learning (ML) is a subset of AI technology that uses data-driven techniques in which algorithms discover and learn patterns and features from data [156], subsequently making predictions or classifications according to the acquired knowledge. Deep learning, a subset of machine learning, involves the construction of high-level abstract models of data through multilayered processing architectures and has been widely applied in tumor diagnosis and prognostic modeling [157]. The integration of machine learning with sequencing technologies represents a major direction in assessing DNA methylation for cancer diagnosis, offering several advantages, such as (1) enhanced diagnostic specificity and sensitivity, where AI models, particularly those employing ensemble learning, integrate signals from multiple methylation biomarkers, reduce noise interference, and minimize false negatives/positives; and (2) precise tissue-of-origin identification, which is frequently achieved through simultaneous cancer classification and tumor origin tracing. Deep learning approaches, such as convolutional neural networks (CNNs), are used to analyze WGBS data. These models decipher the genome-wide methylation profiles, thereby determining the anatomical origin of a tumor. Xin Zhang et al. developed a deep neural network model for pulmonary malignancy discrimination using targeted methylation sequencing of bronchial washing fluid samples. The model achieved 98.6% sensitivity and 97.8% specificity, demonstrating substantial advantages over conventional bronchoscopic diagnostics [158]. A semi-reference-free deconvolution (SRFD) algorithm has been developed to automatically generate reference databases from cfDNA methylation profiles, circumventing manual tissue data curation. Integration of tumor-component features with machine learning classification was implemented using the SRFD-Bayes framework [159]. Validation of late-stage-trained models using early-stage cancer samples achieved early detection sensitivity and specificity rates of 86.1% and 94.7%, respectively. This finding substantiates that machine learning furnishes a robust technical foundation for precision oncology diagnostics.

#### Multimodal learning

Researchers have developed multimodal tumor diagnostic models that integrate methylation data with multisource biomedical data, including genomics and radiomics data, thereby improving the accuracy of cancer diagnosis [160]. The core of multimodal machine learning lies in cross-modal data fusion, which aims to map heterogeneously distributed data from diverse sources and modalities into a unified representation space. This approach effectively overcomes the limitations of unimodal analysis and delivers comprehensive diagnostic insights by capturing complementary information across modalities. A recent review provides a comprehensive overview of multimodal deep learning fusion strategies in cancer applications [161]. Furthermore, a high-performance diagnostic strategy leveraging DNA methylation as a distinct multimodal component has been developed, demonstrating significant clinical validity in empirical validation. Nguyen VTC et al. developed a multimodal approach known as ‘SPOT-MAS’ [162], which enables the simultaneous detection and localization of multiple cancers from a single screening by comprehensively analyzing the methylation profiles, fragmentomics, DNA copy number variations, and terminal motifs of cfDNA. In a study, researchers developed a multimodal classifier using radiological, DNA methylation, and clinical data via the random forest algorithm to predict BRAF mutation status in melanoma patients, achieving an AUC of 0.8 [163]. The essential role of DNA methylation as a core feature within multimodal learning frameworks reveals its extensive diagnostic utility and substantial promise across tumor classification and clinical outcome prediction.

##### Outlook

DNA methylation analysis is increasingly essential for tumor diagnostics, with its impact directly proportional to the rapid evolution of detection methodologies [164,165]. The progression from bisulfite sequencing in first-generation platforms to WGBS in second-generation platforms and now to nanopore sequencing has resulted in substantial advancements. These technologies have increased the sensitivity and expanded the scope of detection, greatly facilitating the precision and efficiency of methylation analyses. We summarize the characteristics of main methylation detection platforms (Table 2). Many challenges and limitations in methylation detection remain to be resolved in clinical applications. First, novel methods for methylation transformation should be established. Bisulfite-based sequencing can cause DNA damage, especially to cfDNA, potentially leading to DNA degradation, which may affect sequencing accuracy. Enzymatic-based mild conversion methods, such as TET and APOBEC3A enzymes, are being investigated to reduce DNA degradation while maintaining high conversion efficiency and specificity. Continuous optimization of enzymatic conversion conditions is necessary and may replace the traditional bisulfite conversion method. Second, detection sensitivity and specificity should be increased. Detecting methylation markers is a feasible approach for early cancer screening and diagnosis. However, some clinical samples, such as cfDNA, are present at low concentrations at early disease stages, which demands greater sensitivity from detection technologies. Third-generation sequencing technology offers a new perspective for methylation detection. Nevertheless, compared with traditional bisulfite sequencing methods, its accuracy requires improvement. The application of machine learning algorithms can effectively increase the accuracy of nanopore sequencing. Additionally, developing novel nanopores is crucial for improving detection accuracy. Third, diverse assay platforms should be explored. Although high-throughput sequencing enables the simultaneous detection of multiple methylation sites, it has several limitations, such as extended processing times and high costs. For specific cancers, various detection technologies utilizing PCR platforms, such as qPCR, dPCR, and sensor assays, can be effectively employed. Consequently, it is necessary to develop more straightforward, convenient, and sensitive methods to meet diverse clinical needs and application scenarios. Fourth, the cost of detection should be reduced. High-throughput sequencing technologies involve expensive sequencing instruments, specific reagents, and complex bioinformatics analysis workflows, resulting in typically high costs, such as WGBS. Targeted sequencing is relatively low in cost, but provides limited information. Furthermore, third-generation sequencing is still in the developmental stage and is similarly costly. Optimizing library preparation and equipment costs and developing automated analysis software can reduce the costs associated with methylation detection. Fifth, large-scale clinical validation should be obtained. The optimization and application of methylation detection technologies are reliable for extensive clinical sample validation. However, the high costs and limitations in sourcing and managing clinical samples restrict opportunities for large-scale multicenter validations. Accordingly, enhancing the accuracy of third-generation sequencing necessitates ongoing sample validation and methodological refinements. There is a need to intensify clinical research and establish standardized detection protocols to advance the broad clinical application of DNA methylation markers. Finally, regarding sample sourcing and processing, fecal DNA extraction from exfoliated cells is more sensitive for early CRC diagnosis, and urine cfDNA methylation is also sensitive for bladder and renal cancer detection. Different samples exhibit specific selectivities for detection methods. For example, cfDNA is characterized by fragmentation and a shorter length, which renders the bisulfite conversion method inappropriate. Enzymatic conversion approaches are more suitable in such cases. Additionally, it is imperative to refine the extraction protocols for cfDNA to ensure its quality and prevent contamination from genomic DNA.

**Table 2.** The characteristics of the methylation detection platforms.

Methods	Qualitative or quantitative	Assessment methylation status	Sensitivity/Specificity	Throughput	Advantages	Disadvantages	Cost	Ref
MSP	Qualitative	All CpGs in the primer or probe	+/+ +	+	Simple operation	Can not single resolution	+	[67]
BSP	Quantitative	One-all CpGs in the amplicon	+ +/+ + +	+	High specificity	Tedious and time consuming operation	++	[77]
Bisulfite Pyrosequen-cing	Quantitative	One-all CpGs in the amplicon	+ +/+ +	++	Rapid and reproducibl-e	Primer design limitation	++	[78]
Bisulfite Sequencing	Quantitative	One-all CpGs in the amplicon	+ + +/+ + +	+ ++	Single resolution	High cost, long duration, DNA damage	+++	[41]
MS-ddPCR	Qualitative	All CpGs in the amplicon	+ + +/+ +	+	High sensitivity	Can not single resolution,	++	[71]
MSRE qPCR	Qualitative	All CpGs in the amplicon	+/+ +	+	Simple operation	Endonuclease-s limination	+	[103]
RRBS	Quantitative	One-all CpGs in the amplicon	+ + +/+ + +	+ +	Single resolution	Endonuclease-s limination	+++	[119]
MSRE/MDRE-seq	Quantitative	One-all CpGs in the amplicon	+ + +/+ +	+ +	Single resolution	Endonuclease-s limination	+++	[122]
EM-seq	Quantitative	One-all CpGs in the amplicon	+ + +/+ + +	+ ++	High resolution, wide GC coverage	High cost, long duration	+++	[126]
TAPS	Quantitative	One-all CpGs in the amplicon	+ + +/+ + +	+ ++	High sequencing quality and coverage	High cost, long duration	+++	[130]
TGS	Quantitative	One-all CpGs in the amplicon	+ +/+ +	+ +	Long reads, fast speed	High cost, complex data processing	+++	[138]

High: + + +, Moderate: + +, Low: +.

Machine learning has shown great promise in tumor diagnosis by processing large amounts of medical data, identifying complex patterns, reducing human error, and improving diagnostic accuracy. Its applications in biological datasets, including genomics, metabolomics, and imaging data, are increasing. It can identify biological markers, discover new diagnostic patterns, integrate multidisciplinary data, and drive medical research and innovation, thus providing strong support for early tumor diagnosis, precision treatment, and prognostic assessment. However, when applied to methylation data, machine learning faces several challenges. Obtaining large-scale, high-quality methylation data is difficult due to the complexity and high cost of methylation detection techniques. Methylation data also requires extensive preprocessing, such as normalization, handling missing values, and addressing batch effects, which can be time-consuming and may introduce biases if not done properly. Additionally, many machine learning models, especially deep learning models, are often considered ‘black boxes’ because of their complex structures and numerous parameters, making it hard to interpret the biological significance of model predictions and to understand the underlying mechanisms of methylation regulation. Researchers will need to continuously improve and optimize algorithms and modeling methods to better address the challenges.

In addition, with the advancement of genomics, the application of gene and genome tests has become widespread both within and outside the clinical setting, and the use of methylation detection kits in clinical diagnostics is also subject to strict regulation. The FDA has set forth requirements for clinical diagnostic methylation detection products, including analytical validity, clinical validity, and clinical utility, and manufacturers must obtain FDA approval to market their products. Previously, the FDA exercised enforcement discretion regarding laboratory-developed tests (LDTs), allowing them to be used in clinical practice without evaluation. However, the FDA has now proposed a new regulatory framework guidance document to strengthen the oversight of LDTs (https://www.genome.gov/sites/default/files/media/files/2024-02/FDA_Oversight_Regulation_Laboratory_Developed_Tests_LDTs.pdf). Meanwhile, the FDA has also issued guidance documents for the regulation of NGS gene tests (https://www.fda.gov/media/99208/download). Table 3 lists some commonly used tumor methylation detection methods that have been approved. Nevertheless, there are challenges in the practical application of methylation detection kits, such as 1) the low sensitivity and accuracy of approved kits, especially when DNA levels in the blood are low in the early stages of tumors; 2) the lack of standardized performance criteria for detection methods, making it difficult to compare different studies; 3) the potential for false positives and false negatives, which may affect the accuracy of clinical diagnosis; and the high cost of detection.

**Table 3.** Commercially available IVD tests.

Test name	Cancer type	Methylation biomarker	Detection method	Sample type	Manufacture	Type of approval
Epi proLung®	Lung cancer	SHOX2, PTGER4	Real-time PCR	Plasma	Epigenomics AG	CE (2017)
Epi proColon®	Colorectal cancer	SEPT9	Real-time PCR	Plasma	Epigenomics AG	FDA (2016)
EarlyTect® Colorectal Cancer	Colorectal cancer	SDC2	Real-time PCR	Plasma	Epigenomics AG	CE (2017)
HCCBloodTest	Hepatocellular carcinoma	Septin 9	Real-time PCR	Plasma	Epigenomics AG	CE (2019)
Therascreen PITX2 RGQ	Breast cancer	PITX2	Methylation specific real-time PCR	Tissue	QIAGEN GmbH	CE (2018)
UriFind test	Bladder Cancer	ONECUT, VIM	Real-time PCR	Urine	AnchorDx	FDA (2021)
Cervi-M	Cervical cancer	PAX1	Methylation-specific real-time PCR	Cervical smear	EpiGene Bio	FDA (2021)
GynTect®	Cervical cancer	ASTN1, DLX1, ITGA4, RXFP3, SOX17, ZNF671	Methylation-specific real-time PCR	Cervical smear	Oncognostic GmbH	CE (2019)
ConfirmMDx	Prostate cancer	GSTP1, RASSF1, APC	Multiplexed quantitative DNA methylation-specific PCR	Prostate biopsy	MDxHealth	CE (2012)
MGMT Methylation Detection Kit	Glioblastoma	MGMT	Real-time PCR	Plasma	Epigenomics AG	CE (2018)

In conclusion, as research progresses and technology advances, DNA methylation markers hold substantial promise for use in tumor diagnostics. These markers are expected to improve the pursuit of precision medicine in oncology. Future research will continue to explore the potential of these markers while refining detection technologies to improve their accuracy and reliability in clinical practice.

## Data Availability

The data used to support the study are available online.

## Abbreviations

5mC: 5-methylcytosine
5hmC: 5-hydroxymethylcytosine
CGIs: CpG islands
NGS: next generation sequencing
ctDNA: circulating tumor DNA
PBMCs: peripheral blood mononuclear cells
ESCC: esophageal squamous cell carcinoma
MSP: Methylmion Specific PCR
MS-ddPCR: Methylation-specific droplet digital PCR
LAMP: loop-mediated isothermal amplification
BSP: Bisulfite sequencing PCR
WGBS: Whole Genome Bisulfite Sequencing
MSRE/MDRE: Methylation-sensitive/-dependent restriction enzymes
RRBS: Reduced representation bisulfite sequencing
EM-seq: Enzymatic-based DNA methylation sequencing
TET: methylcytosine dioxygenase
APOBEC3A: apolipoprotein B mRNA editing catalytic polypeptide-like 3A
T4-BGT: T4 β-glucosyltransferase
TAPS: TET-assisted Pyridine Borane Sequencing
wgTAPS: whole-genome TAPS
rrTAPS: reduced representation TAPS
eeTAPS: endonuclease enrichment TAPS
DM-Seq: Direct Methylation Sequencing
TGS: Third-generation sequencing
SMRT: single-molecule real-tim
ZMWs: zero-mode waveguides
